# Neglected for Too Long: Perinatal Mental Health Impacts of Stillbirth in Low‐ and Middle‐Income Countries

**DOI:** 10.1111/1471-0528.18051

**Published:** 2024-12-16

**Authors:** Hannah Blencowe, Oona Campbell, Toma Kerac, Renae Stafford, Vandana Tripathi, Veronique Filippi

**Affiliations:** ^1^ London School of Hygiene and Tropical Medicine London UK; ^2^ Univerity of Oxford Oxford UK; ^3^ EngenderHealth Washington DC USA

**Keywords:** depression, low‐ and middle‐income countries, mental health, neonatal death, stillbirth

Stillbirths are one of the world's most neglected tragedies. The estimated 1.9 million babies that are stillborn after 28 completed weeks of pregnancy in 2021 underestimates the overall burden of all fetal deaths from 22 weeks onward [1]. Each death has an important impact on affected women, families and healthcare workers [2]. Yet, despite this, until very recently, national governments, UN organisations and civil society institutions (including non‐governmental organisations and professional associations) worldwide have given little attention to stillbirths. This has resulted in lack of prioritisation of stillbirths in data strengthening efforts, limiting visibility of underlying biomedical causes [3]. This coupled with the failure to include stillbirths in maternal and child health dialogues exacerbates stigma towards affected women, and frequently leaves bereaved parents to deal with their grief in silence.

Perinatal mental health disorders, occurring during pregnancy and in the first year after childbirth, affect almost one in five women giving birth and have only recently been brought out of the shadows [4]. Many organisations are now accelerating their focus on the issue and global guidance on the integration of perinatal mental health into routine healthcare provision is now available [5]. However, despite a strong body of evidence describing increased risks of adverse perinatal mental health outcomes associated with stillbirth in high‐income countries [2], and newer evidence reporting similar effects across a wide range of low‐ and middle‐ income countries (LMICs) [6, 7, 8, 9], stillbirths have been to‐date largely left out of perinatal mental health guidance.

We conducted a rapid review of evidence on the perinatal mental health consequences of stillbirth as part of the MOMENTUM Safe Surgery in Family Planning and Obstetrics (MOMENTUM Safe Surgery) project's work to improve postnatal services [10, 11]. Collating available data across 20 studies from 12 low‐ and middle‐income countries, a median of 41% of women reported evidence of depression in the year following a perinatal loss (stillbirth or early neonatal death) [9]. This is at least double the risk compared to after a live birth, translating to around 1.2 million women annually with depression in the year following a stillbirth or early neonatal death in sub‐Saharan Africa and South Asia alone. Increases in anxiety and stress were also observed, presenting a substantial burden on women, families and communities in these regions.

Adverse perinatal mental health consequences following stillbirth can be partly mitigated through timely, person‐centred supportive bereavement care. The past decade has seen large advances in understanding the experiences and care needs for affected women, partners and families, including the introduction of national care bereavement pathways in several high‐income countries [12]. While the principles for bereavement care are likely to be similar across settings, there are large gaps in evidence in how these can be optimally delivered and tailored to bereaved women in LMICs [13]. In these regions, stillbirth often carries a significant stigma, expressions of grief vary widely, and there may be constraints on frontline health resources. These unique contextual factors highlight the need for targeted research and strategies to address the specific challenges associated with stillbirth bereavement in LMICs.

The MOMENTUM Safe Surgery review looked at interventions to address the perinatal mental health needs of women experiencing stillbirth in LMICs [10]. Only three published small studies (from India and Iran) were located, providing low‐quality evidence of some improved psychological outcomes with mindfulness, individual and small‐group counselling interventions for bereaved women [14, 15, 16]. Three additional studies are currently underway [17, 18, 19].

This lack of a robust evidence base for interventions in LMICs may partly explain the absence of this important issue from global guidance. However, this silence in guidance and training documents potentially misses important opportunities to translate what is currently known into practice. Emerging evidence from the literature shows that women from a wide range of LMIC geographies express a shared desire for specific components of care aimed at enhancing their well‐being after stillbirth. Notably, communication, active involvement in management decisions, having physical needs met, receiving respectful care and personalised support for subsequent pregnancies are consistently identified as crucial themes in improving the overall experience for these women (see Box 1) [10].

BOX 1 **Positive aspects of care reported by women to improve experience and well‐being after stillbirth in LMICs [10].**

*Communication*:

*Breaking bad news*: Communicate truthfully, clearly and compassionately to the woman directly that her baby has died. In her own language. In a private space. Check understanding.

*Ongoing communication*: Offer to communicate to family members afterwards if woman desires. Show empathy and sensitivity to the woman and family's needs and preferences. Verbal encouragement/ comfort. Encourage questions.

*Around cause of death*: Provide the most accurate information on cause of death available, including death certification where possible.^a^ Attributing death to a higher power, ‘God's will’, may not be appreciated by all.

*Involvement in management decisions*: Provide opportunity to discuss management options for delivery and postnatal care, including decisions around sedation and suppression of lactation. Avoid paternalistic decision‐making on behalf of woman or defensive behaviours (to avoid blame).

*Having physical needs met*: Optimal management of physical condition, both around the time of childbirth including appropriate analgesia and ongoing post‐partum care.

*Respectful care*: Provision of option for care separated from women with live births. Provision for presence of family if desired throughout care. Respectful handling of the stillborn baby. Provide opportunity to see and hold baby if desired.

*Tailored care for next pregnancy*: To address physical and emotional needs, tailored to women's preferences and taking into account the cause of previous stillbirth.

^a^Where there is a lack of information on cause of death women left to construct their own explanation, enforcing cultural misbeliefs around supernatural causes of stillbirth.

In addition to the increased psychological risks, women experiencing stillbirth are also known to be at higher risk of physical complications, such as post‐partum haemorrhage, infection and obstetric fistula. Current normative standards for postnatal care developed by the World Health Organization focus on ‘uncomplicated’ births only, a missed opportunity to intervene early to prevent, detect and manage both physical and psychological complications and provide ongoing care such as voluntary family planning services and planning care for next pregnancy for those experiencing birth complications, including stillbirth [20]. Action is needed to develop extensions to the existing postnatal care guidelines to provide best practice guidance for the ongoing care of bereaved women. In addition, as routine postnatal care is often perceived to revolve around the care and immunisation of the baby, bereaved women frequently have lower levels of engagement despite their more complex needs. Therefore, more pro‐active approach may be required, both to facilitate engagement with the health services in this critical period and to provide tailored care to ensure the needs of this population are met.

In addition to the 1.9 million women experiencing stillbirth worldwide each year, a further 1.7 million will be affected by early neonatal death. As many of these deaths are preventable, action to improve timely, equitable access to high‐quality care along the continuum is urgently needed, but must be coupled with action to improve the supportive care that bereaved women receive to reduce psychological morbidity. There are many similarities between the experiences of women affected by stillbirth and early neonatal deaths [21]. A large proportion of these deaths occur in health facilities, with the babies dying before birth or before leaving the health facility. In these cases, the baby's existence or personhood is rarely being acknowledged, names seldom given, most never are introduced to the wider family, cultural events such as burials infrequently undertaken; hence their births and deaths are surrounded with stigma [22].

Summarising the evidence is a critical first step towards including the specific needs of bereaved women and fathers into global and national guidance and training materials. Including bereaved women, husbands, partners, family and the wider community alongside health workers in future intervention research will be essential across a wide range of contexts. Further research to close specific evidence gaps may be needed. For example, the impact of stillbirth on partners, children and other family members in many settings is poorly understood. In addition, although healthcare workers such as obstetricians and midwives are the main providers of clinical and supportive care for women and families around the time of stillbirth, the high emotional demands of this role alongside regular duties are frequently overlooked. Future studies are needed to understand the needs of health workers around the time of a stillbirth and how their support can best be integrated into comprehensive bereavement care provision.

Going forward, existing and emerging evidence in this area can be used together with examples of best practice to improve provision of supportive care for every bereaved mother. There is an urgent need for normative guidance upon principles for supportive care after a stillbirth or neonatal death based on existing evidence and widespread stakeholder consultation including UN partners, professional associations, frontline health professionals and bereaved parents with high representation from high‐burden settings. These will need revising as new evidence emerges.

And whilst this process is undertaken, what is increasingly clear is that we have sufficient evidence to implement three important changes now. First, we can increase awareness among communities of healthcare professionals around the specific issues faced by women following stillbirth and early neonatal death. Second, provision of communication training and support to those healthcare professionals caring for affected women is possible now in all settings. National and international organisations of obstetricians and midwives can play a critical role in achieving this. Thirdly, societal awareness and understanding about stillbirths and early neonatal deaths in every country could be increased for example through information and media campaigns. Implementing these steps now could ultimately improve perinatal mental health and well‐being among bereaved mothers everywhere.

## Author Contributions

H.B. drafted the initial draft of the commentary. H.B. and T.K. undertook the review and synthesis of the findings relating to the stillbirth impact of perinatal mental health. All authors reviewed and refined the draft. All authors approved the submitted version.

## Ethics Statement

No primary data were collected as part of this commentary. Ethical permissions were not required.

## Conflicts of Interest

The authors declare no conflicts of interest.

## Data Availability

Data sharing not applicable to this article as no datasets were generated or analysed during the current study.
